# Salvianolic Acid B Inhibits Ferroptosis in Acute Lung Injury: Network Analysis and Experimental Validation

**DOI:** 10.1111/jcmm.71344

**Published:** 2026-09-03

**Authors:** Hejun Gao, Renhui Xiong, Na Yue, Jiaxin Wang, Shouyuan Tian

**Affiliations:** ^1^ Shanxi Provincial People's Hospital Affiliated to Shanxi Medical University Shanxi Provincial People's Hospital, The Fifth Clinical Medical College of Shanxi Medical University Taiyuan China; ^2^ Shanxi Key Laboratory of Geriatric Precision Anesthesia and Complication Prevention Shanxi Provincial People' Hospital Taiyuan China; ^3^ Cancer Center Daping Hospital & Army Medical Center of PLA; Army Medical University (Third Military Medical University) Chongqing China; ^4^ Department of Anesthesiology Ordos Central Hospital Ordos China; ^5^ Department of Anesthesiology The First Medical Center of Chinese PLA General Hospital Beijing China

**Keywords:** acute lung injury, ALB, ferroptosis, PI3K/AKT signalling pathway, salvianolic acid B

## Abstract

This current study aimed to decipher the anti‐inflammatory, anti‐ferroptosis and pronounced lung‐protective activities of Salvianolic acid B (Sal B) by integrating network pharmacology and experimental confirmation across multiple aspects. The optimal target protein and the relevant molecular mechanisms for Sal B in the context of ALI were analysed using network pharmacology. Molecular dynamics simulations and BLI were performed on the core protein ALB–compound Sal B identified through molecular docking. The pharmacological efficacy of Sal B and its underlying mechanisms were further substantiated through both in vitro and in vivo experiments, utilizing the LY294002 inhibitor and si‐ALB to confirm the involvement of ALB as a target. Additionally, RNA sequencing of murine lung tissues was conducted to further elucidate the molecular mechanism of Sal B. The results of network pharmacology indicated that a total of 155 potential target genes through which Sal B may exert its protective effects against ALI were identified, with essential pathways including PI3K‐AKT, TNF and IL‐17 signalling pathways. Molecular docking, MD, and BLI demonstrated stable binding between Sal B and the core target ALB. In vitro and in vivo evidence confirmed that Sal B attenuates LPS‐induced ALI by inhibiting ferroptosis via the PI3K/AKT axis, as corroborated by LY294002 and si‐ALB intervention. RNA sequencing and network pharmacological analysis synergistically revealed the PI3K/AKT pathway as a potential signalling pathway for Sal B. Sal B ameliorates sepsis‐induced ALI via a defined mechanism that involves the activation of the PI3K‐AKT pathway, thereby inhibiting ferroptosis both in vivo and in vitro.

## 1 Introduction

Acute lung injury (ALI), a prevalent and serious complication of sepsis, is defined as a non‐cardiogenic acute respiratory disorder, which is triggered by increased alveolar‐capillary permeability, manifesting as rapid‐onset hypoxemia and bilateral pulmonary edema, and can escalate to acute respiratory distress syndrome (ARDS) if not promptly treated [1, 2, 3, 4]. This disease poses a significant global health burden due to its high incidence and mortality rates worldwide [5]. Current ALI management remains supportive, including pharmacological and cell‐based strategies, with no specific disease‐modifying therapy yet available [6]. Consequently, there is an urgent need for the development of novel therapeutic strategies for sepsis and the exploration of potential treatments for ALI.

Derived from *Salvia miltiorrhiza* , Sal B is characterized as a water‐soluble phenolic acid and represents one of its principal bioactive constituents [7]. Recognized as one of the most potent antioxidant compounds among known phenolic acids, this molecule is structurally comprised of an ester formed from the condensation of three danshensu molecules with one caffeic acid unit [8]. In addition, Sal B demonstrates multifaceted pharmacological activities through distinct yet interconnected mechanisms. First, Sal B exhibits significant antioxidant activity by directly scavenging free radicals and inhibiting lipid peroxidation [9]. Also, it confers protection against myocardial ischemia–reperfusion injury via anti‐inflammatory, anti‐apoptotic, and microcirculation‐improving mechanisms [10, 11, 12]. Additionally, Sal B inhibits fibrotic processes in organs such as the liver and kidneys [13, 14]. Notably, Sal B has shown promising clinical potential in ALI management. Sal B, through the suppression of apoptosis, oxidative stress, and inflammation, significantly ameliorates core pathological features in septic rats [15, 16]. It could ameliorate ALI in septic rats by downregulating the channel kinases TRPM6 and TRPM7 [17]. Despite this promise, the specific effects and mechanisms of Sal B in ALI require further clarification.

The PI3K/AKT pathway is a pivotal regulator of ferroptosis, coordinating this form of cell death by modulating key components such as NRF2, GPX4, SLC7A11, Fe^2+^, and lipid metabolism [18]. Prior research has demonstrated that the activation of this pathway, including the PI3K/AKT/NRF2 and PI3K/MAPK/AKT axes, or through the upregulation of SLC7A11 and GPX4, can suppress ferroptosis [19, 20, 21]. In the situation of ALI, the activation of the PI3K/AKT pathway confers comprehensive cellular protection, which is characterized by the concerted attenuation of inflammation, suppression of apoptosis, and inhibition of ferroptosis. Thus, this pathway may be a promising mechanism for ALI therapy.

**FIGURE 1:**
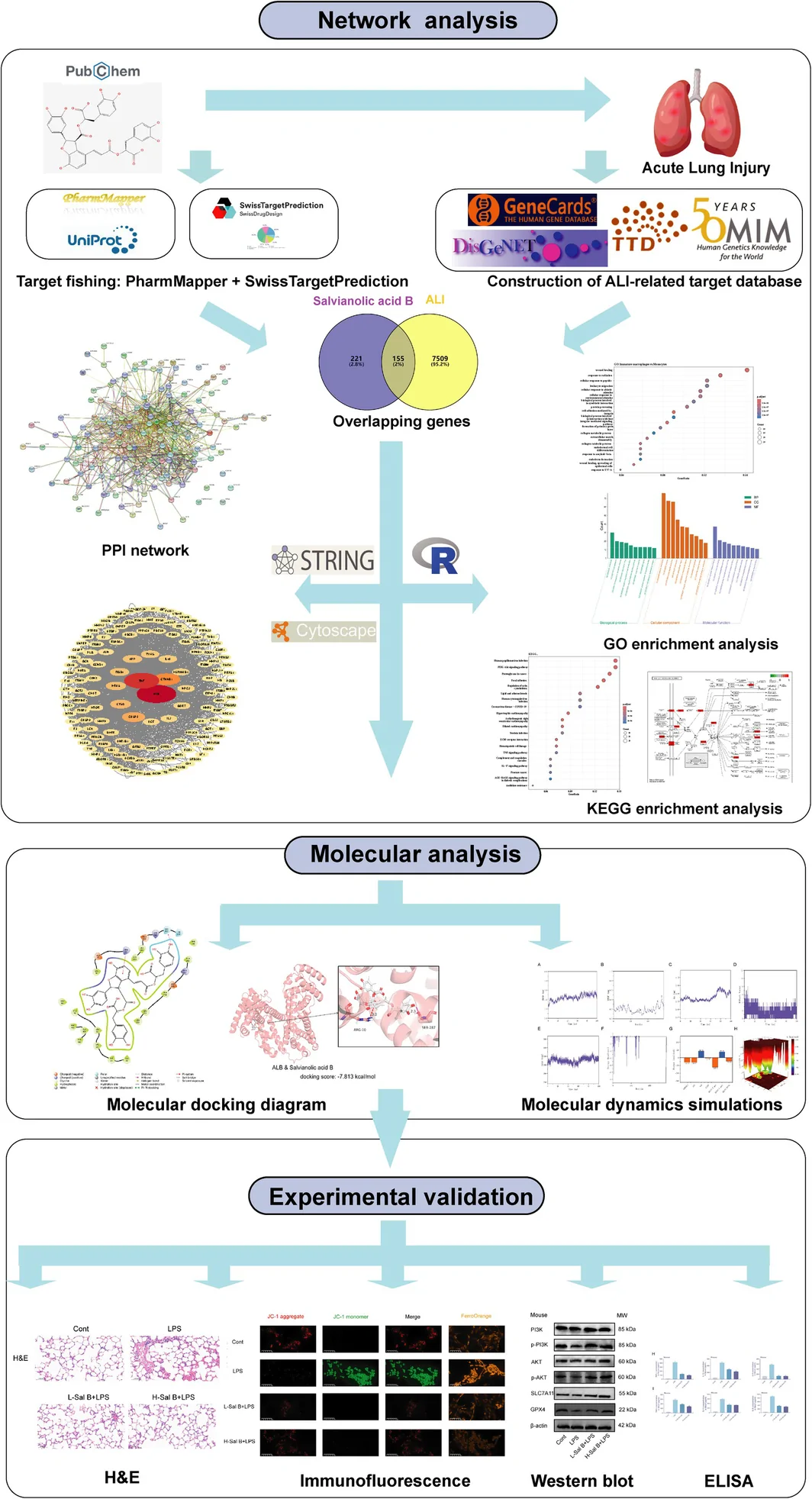
Workflow of the network pharmacological study of Sal B in treating ALI.

In this study, we elucidate a new insight based on network pharmacology combined with experimental validation for the protective effects of Sal B against LPS‐induced ALI, thereby providing a theoretical basis for its drug development in the future. A schematic diagram summarizing this integrated strategy is provided in Figure 1.

## 2 Materials and Methods

### 2.1 Identification of Sal B Targets

The identification of Sal B targets was initiated by retrieving its 2D structure from PubChem (https://pubchem.ncbi.nlm.nih.gov/). This structure was subsequently submitted to the PharmMapper platform (http://www.lilab‐ecust.cn/pharm‐mapper/) for reverse pharmacophore matching and to the SwissTargetPrediction database (http://www.swisstargetprediction.ch/) for target profiling, in parallel. Potential targets identified from both platforms were consolidated, and their union was established to generate a comprehensive set of putative biological targets for Sal B.

### 2.2 Identification of ALI‐Related Targets

ALI‐related targets were obtained as follows: previous research [22].

### 2.3 The Antagonistic Targets of Sal B Against ALI

To identify the intersecting targets, the Sal B target set (Section 2.1) and the ALI target set (Section 2.2) were input into the Venny 2.1 online platform (https://bioinfogp.cnb.csic.es/tools/venny/). The overlapping targets identified through this visualization were designated as candidate targets for Sal B against ALI and used for subsequent analysis.

### 2.4 PPI Network Construction

The previously identified cross‐target genes were analysed and PPI network was constructed as described in previous research [22].

### 2.5 Functional Enrichment Analysis

The overlapping Sal B‐ALI targets from Section 2.3 were functionally annotated via the DAVID database (https://david.ncifcrf.gov/) [23, 24]. The significant results were subsequently visualized using the Bioinformatics online platform (http://www.bioinformatics.com.cn/).

### 2.6 Molecular Docking

The 3D structure of Sal B and ALB were obtained from PubChem/PDB, followed by format conversion (OpenBabel), molecular docking (AutoDock Tools 1.5.7), and visualization (Discovery Studio 2021) [22].

### 2.7 Molecular Dynamics Simulation

The molecular dynamics (MD) simulations of Sal B with ALB were performed using the GROMACS 2022 software. Specific methods were as previously described [22].

### 2.8 Bio‐Layer Interferometry Assay

Recombinant human (rh) ALB was biotinylated and desalted to remove the unbound biotin. Subsequently, the biotinylated rhALB was specifically captured and diluted to a total volume of 400 μL using PBS, employing Super Streptavidin Biosensors (Sartorius, Goettingen, Germany), and immobilized for 3000 s. Upon reaching a signal of 4.5 nm, it was exposed to different concentrations of Sal B. The Octet R8 Protein Analysis System (Sartorius, Goettingen, Germany) was utilized to measure binding and dissociation rates, enabling the calculation of the protein–small molecule affinity.

### 2.9 Drugs

LPS ( *Escherichia coli* 055:B5) (Sigma, St Louis, MO, USA). Sal B (Manster Biotechnology Co. Ltd., Chengdu, China). LY294002 (MedChemExpress, Shanghai, China). Primary antibodies used were as follows: rabbit anti‐SLC7A11 (1:1000, Proteintech Group, Wuhan, China), mouse anti‐GPX4 (1:3000, Proteintech Group, Wuhan, China), mouse anti‐β‐actin (1:10000, Proteintech Group, Wuhan, China), rabbit p‐PI3K (1:1000, Abcam, Shanghai, China), rabbit anti‐p‐AKT (1:2000, Abcam, Shanghai, China), rabbit PI3K (1:1000, Abcam, Shanghai, China), rabbit anti‐AKT (1:2000, Abcam, Shanghai, China).

### 2.10 Animals

Male C57BL/6 mice (8–12 weeks old) were sourced from the Experimental Animal Center of Shanxi Provincial People's Hospital. All protocols received approval from the Medical Ethics Committee of Shanxi Provincial People's Hospital (No. 2025–611). First, the purchased mice were allowed to acclimatize for 7 days, and their weight and behavioural status was recorded daily. After confirming that there were no abnormalities, gastric treatment was carried out: The mice were divided into four groups (*n* = 5) following a one‐week acclimatization: (1) control, (2) LPS, (3) L‐Sal B (10 mg/kg) + LPS (4 mg/kg), and (4) H‐Sal B (20 mg/kg) + LPS. At 4 h post‐administration of LPS or 100 μL PBS via intranasal instillation (Figure 6A). After anaesthesia with isoflurane and analgesia with buprenorphine, eye socket blood was collected. Immediately after blood collection, the mice were euthanized by cervical dislocation and confirmed to have dilated pupils and cardiac arrest. Finally, the collected blood was subjected to sample processing: the whole blood was left to stand at 4°C for 30 min, then centrifuged at 3000 rpm for 15 min in a centrifuge pre‐cooled to 4°C. The separated serum was packaged and frozen in liquid nitrogen before being transferred to a −80°C ultra‐low‐temperature freezer for long‐term storage.

### 2.11 BALF Collection

Bronchoalveolar lavage was performed in mice by instilling and withdrawing 1 mL of PBS through a tracheal cannula, repeated three times. The collected BALF was then centrifuged at 3000 rpm for 10 min at 4°C. The resulting cell pellet was resuspended in 0.1 mL of PBS for cell counting, while the supernatant was aliquoted for protein concentration measurement using a BCA assay (Beyotime, Shanghai, China) and subsequent analysis of inflammatory factors.

### 2.12 Determination of Lung Wet/Dry (W/D) Weight Ratio

Following collection, lung samples were immediately weighed to obtain the wet weight and then placed in a 60°C oven for 72 h to determine the dry weight. The lung wet/dry (W/D) ratio was calculated to assess the degree of pulmonary edema.

### 2.13 Haematoxylin‐Eosin (H&E) Staining and Pathological Assessment

H&E staining was performed on lung tissue sections to assess the extent of pulmonary injury. A standardized scoring system (range 0–4) was applied to quantify histopathological alterations: a score of 0 indicated normal alveolar architecture; 1 reflected diffuse alveolar wall reactions in the absence of interstitial thickening; 2 represented diffuse inflammatory cell infiltration accompanied by mild interstitial thickening; 3 denoted moderate interstitial thickening with evident inflammatory infiltration; and 4 was assigned when interstitial thickening occupied more than 50% of the microscopic field. The final histopathological score for each sample was expressed as the mean value calculated from three randomly selected fields.

### 2.14 Cell Culture and Model Establishment

Human normal lung epithelial cells (BEAS‐2B) were obtained from Beyotime (C6106, Shanghai, China). Approximately 1 × 10^6^ cells/mL were seeded into six‐well plates before treatment. LY294002 (5 μM) was administered 2 h before Sal B (5 μM, 10 μM) treatments for 24 h, followed by the addition of LPS (20 μg/mL) to each well for 24 h. All cells were maintained in Dulbecco's Modified Eagle Medium (DMEM) supplemented with 10% fetal bovine serum and 1% penicillin–streptomycin antibiotic mixture. The DMEM product (catalogue number: 319–005‐CL) was sourced from WISENT, and it is formulated with 4.5 g/L D‐Glucose, L‐Glutamine, phenol red, and sodium pyruvate. All cultures were incubated in a humidified incubator with a stable atmosphere of 5% CO_2_ at a constant temperature of 37°C.

### 2.15 MPO Activity Detection

The levels of MPO in lung tissue homogenates were assessed according to the instructions of a commercial kit (Nanjing Jiancheng Bioengineering Institute, Nanjing, China).

### 2.16 Enzyme‐Linked Immunosorbent Assay (ELISA)

Levels of TNF‐α, IL‐1β, and IL‐6 were determined in BALF, serum, and cell supernatant using commercial ELISA kits from Solarbio (Beijing, China).

### 2.17 RNA Extraction and Real‐Time PCR

The mRNA expression levels of TNF‐α, IL‐1β, and IL‐6 were determined by quantitative PCR (qPCR). Briefly, total RNA was isolated using TRIzol reagent (Takara‐Bio, Japan) and reverse‐transcribed into cDNA. qPCR reactions were carried out using SYBR Mix, with specific primer sequences provided in Table S1.

### 2.18 Western Blot Analysis

Protein extracts from lung tissues and BEAS‐2B cells were prepared using RIPA lysis buffer. The specific steps of western blotting are as previously described [25]. Briefly, membranes were incubated with primary antibodies against p‐PI3K, p‐AKT, PI3K, AKT, SLC7A11, GPX4, and β‐actin overnight at 4°C. The membrane was detected by using ECL (Thermo) for the visualization of antibody binding.

### 2.19 Assay of MDA, SOD, and GSH

Prepared lung tissue homogenates and cell lysates were used for subsequent analysis. The concentrations of MDA, SOD, and GSH were quantified according to the manufacturer's protocols using commercial assay kits (Beyotime, Shanghai, China).

### 2.20 JC‐1 Staining

To assess mitochondrial membrane potential, BEAS‐2B cells subjected to LPS stimulation for 24 h with or without Sal B pretreatment were stained with the JC‐1 probe (Beyotime, Shanghai). After a 30‐min incubation at 37°C, cells were thoroughly washed with JC‐1 buffer and imaged under an Olympus DX51 fluorescence microscope (Tokyo, Japan). The red/green fluorescence ratio was calculated to determine the degree of mitochondrial impairment.

### 2.21 Detection of Fe^2+^

Intracellular iron accumulation in BEAS‐2B cells was assessed using two steps. First, an iron staining kit (Boxbio, Beijing, China) was used according to the manufacturer's protocol. Following this, cells were incubated with the FerroOrange probe (Dojindo, Japan) at 37°C for 30 min. Fluorescence images from both assays were captured using a fluorescence microscope.

### 2.22 RNA Sequencing

RNA sequencing (RNA‐Seq) profiling of lung tissue samples was conducted by Shenzhen SMo Group Medical Laboratory. Total RNA was extracted using TRIzol reagent. Approximately 3 μg of this RNA was used to construct transcriptome libraries with the TruSeq RNA Sample Preparation Kit (Illumina). Sequencing was performed on an Illumina NovaSeq 6000 system. For library construction, cDNA was synthesized from the RNA using the SuperScript Double‐Stranded cDNA Synthesis Kit (Invitrogen) and random hexamer primers (Illumina). Differential expression analysis identified genes meeting the significance threshold (|log_2_ FC| > 1.5, adjusted *p*‐value < 0.05). Functional enrichment analysis of these differentially expressed genes (DEGs) was performed using the DAVID Bioinformatics database with Wiki Pathways annotation.

### 2.23 Cell Transfection

The ALB small interfering (si)RNA and negative control (NC) sequences were designed and synthesized by Genomeditech (Shanghai) co. Ltd. The siRNA sequences were as follows: mmu‐ALB‐si‐1: GGAGAAGCAGAUUAAGAAACATT, UGUUUCUUAAUCUGCUUCUCCTT; mmu‐ALB‐si‐2: GCUGAGACUUGCUAAGAAAUATT, UAUUUCUUAGCAAGUCUCAGCTT; mmu‐ALB‐si‐3: GCUGAGCAGUACAAUGAGAUUTT, AAUCUCAUUGUACUGCUCAGCTT. Cell transfection was performed according to the manufacturer's instructions for Lipofectamine 3000 (Invitrogen; Thermo Fisher Scientific Inc.). The final concentration of siRNA was 20 nM. After 6 h of culture at 37°C in a humidified 5% CO_2_ incubator, the transfection medium was replaced with fresh complete culture medium. Subsequent experiments were performed after 36 h.

### 2.24 Statistical Analysis

Data analysis was performed using GraphPad Prism 8, with results presented as mean ± SEM. The research followed a randomized, blinded approach without excluding any data points. For comparing two groups, we applied Student's *t*‐test, whereas multiple group comparisons were evaluated through one‐way ANOVA and subsequent Tukey's post hoc analysis. Statistical significance was established at a *p*‐value below 0.05.

## 3 Results

### 3.1 Prediction and Identification of Sal B‐ALI Targets

**FIGURE 2:**
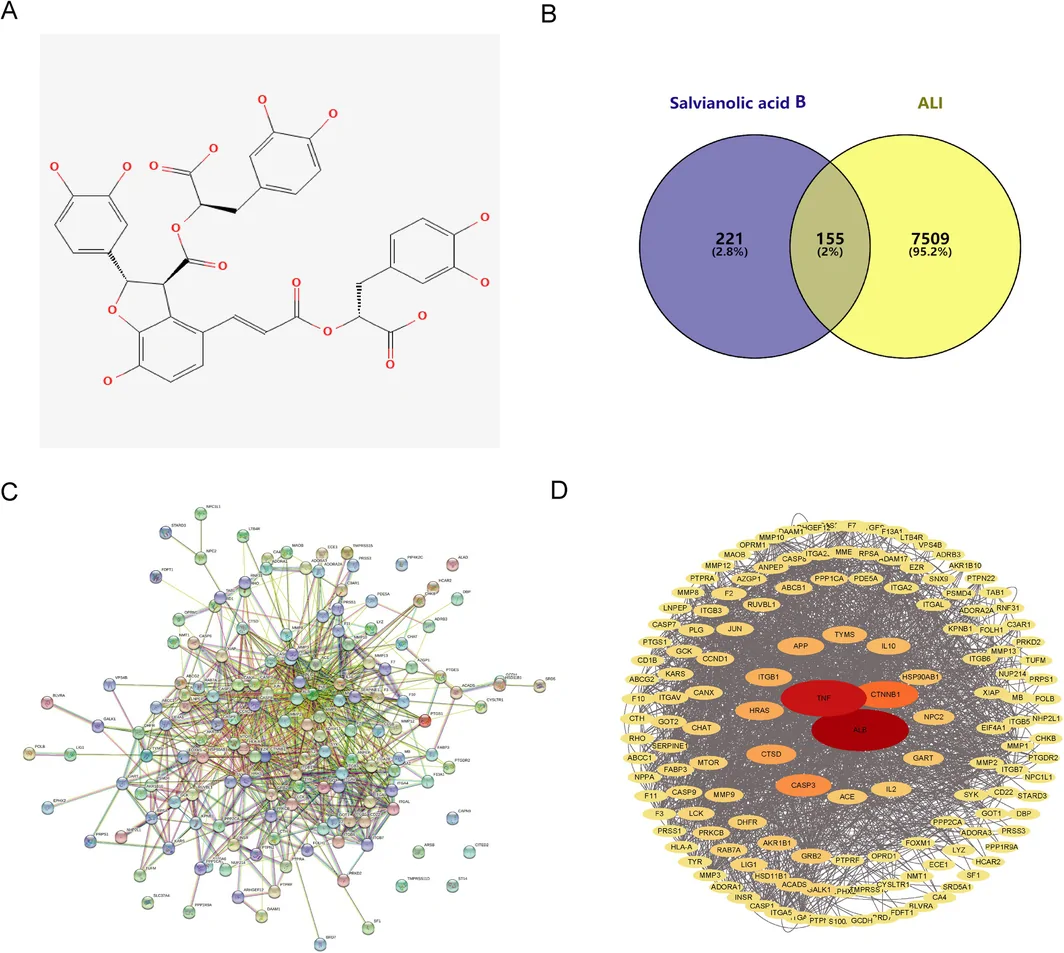
Identification of targets of Sal B‐ALI. (A) Molecular formula of Sal B. (B) Venn diagram of intersecting targets of Sal B in treating ALI. (C) Primary PPI network. (D) PPI network.

Initially, the 2D structure of Sal B was retrieved and yielded 101 potential targets (Figure 2A). Simultaneously, target profiling from the SwissTargetPrediction database identified 276 putative targets. Following the consolidation of results from both platforms, a combined set of 376 non‐redundant targets was established for subsequent analysis. Database mining of GeneCards, OMIM, DisGeNet, and TTD yielded 2033 ALI‐associated targets, which were consolidated into 1809 unique genes after deduplication. Subsequent intersection of the above predicted targets using the Venny diagram identified 155 overlapping genes as the core candidate targets for Sal B against ALI (Figure 2B).

### 3.2 PPI Network Analysis

The shared Sal B‐ALI targets were employed for PPI analysis (Figure 2C). As depicted in Figure 2D, node colour intensity reflects the degree of connectivity. Topological analysis identified the top 30 hub genes, with the core network comprising key targets such as ALB, TNF, CTNNB1, CASP3, CTSD, HRAS, ITGB1, APP, TYMS, and IL10 (Table S2).

### 3.3 Function Enrichment Analysis

**FIGURE 3:**
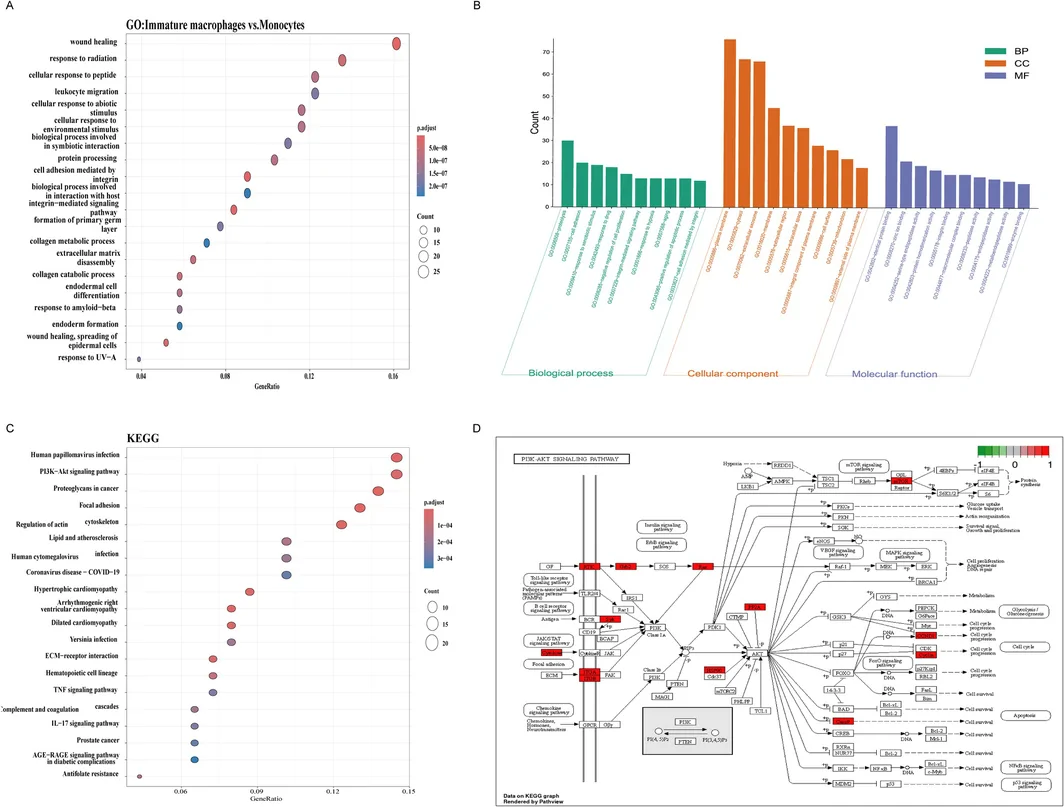
Functional enrichment analysis of potential targets of Sal B for ALI treatment. (A) The bubble chart of the top 20 terms based on GO enrichment analysis. The X‐axis and Y‐axis show the gene ratios and full names of the processes, respectively, and the colour and size of each bubble represent the *p* value and gene count. (B) Top 10 BP terms, cellular CC terms, and MF terms of GO enrichment analysis are shown as green, orange, and purple bars, respectively. (C) The bubble chart of the top 20 pathways based on KEGG enrichment analysis. The size of the bubble is proportional to the number of enriched genes in the term, and the intensity of the red colour corresponds to the level of statistical significance, with a deeper red hue indicating a lower *p*‐value. (D) PI3K‐AKT signalling pathways of potential target genes of Sal B in treating ALI.

A total of 345 significant GO terms were identified (*p* < 0.05), which were categorized into 213 biological processes (BP), 63 molecular functions (MF), and 69 cellular components (CC). The top 10 most significantly enriched terms from each category, ranked by *p*‐value, are presented in the bar chart. Analysis of the BP category revealed predominant involvement in proteolysis, cell adhesion, response to xenobiotic stimulus, response to drug, negative regulation of cell proliferation, integrin‐mediated signalling pathway, response to hypoxia, aging, positive regulation of apoptotic process, and integrin‐mediated cell adhesion. The CC analysis revealed significant enrichment in the plasma membrane, cytosol, extracellular exosome, membrane, extracellular region, extracellular space, integral component of plasma membrane, cell surface, mitochondrion, and external side of plasma membrane. MF analysis indicated predominant involvement in identical protein binding, zinc ion binding, serine‐type endopeptidase activity, protein homodimerization activity, integrin binding, macromolecular complex binding, peptidase activity, endopeptidase activity, metalloendopeptidase activity, and enzyme binding (Figure 3A, 3B).

KEGG pathway analysis indicated that the 155 overlapping targets were significantly enriched in 88 pathways (*p* < 0.05). The top 20 pathways, ranked by *p*‐value, are displayed in a bubble chart based on enrichment significance (Figure 3C). These include: human papillomavirus infection, PI3K‐AKT signalling pathway, proteoglycans in cancer, focal adhesion, regulation of actin cytoskeleton, lipid and atherosclerosis, human cytomegalovirus infection, coronavirus disease COVID‐19, hypertrophic cardiomyopathy, arrhythmogenic right ventricular cardiomyopathy, dilated cardiomyopathy, Yersinia infection, ECM‐receptor interaction, haematopoietic cell lineage, TNF signalling pathway, complement and coagulation cascades, IL‐17 signalling pathway, prostate cancer, AGE‐RAGE signalling pathway in diabetic complications, and antifolate resistance (Figure 3C). Notably, the PI3K‐AKT signalling pathway ranked as the second most significantly enriched. Then, we highlighted 13 key targets (marked in red) within this pathway that are potentially involved in Sal B's therapeutic effects against ALI (Figure 3D). The enriched pathways are predominantly implicated in pivotal processes such as apoptosis, proliferation, oxidative stress, and inflammation. This collective profile suggests that the therapeutic effect of Sal B on sepsis‐induced ALI is likely mediated through the concerted regulation of multiple pathways.

### 3.4 Molecular Docking and Bio‐Layer Interferometry

**FIGURE 4:**
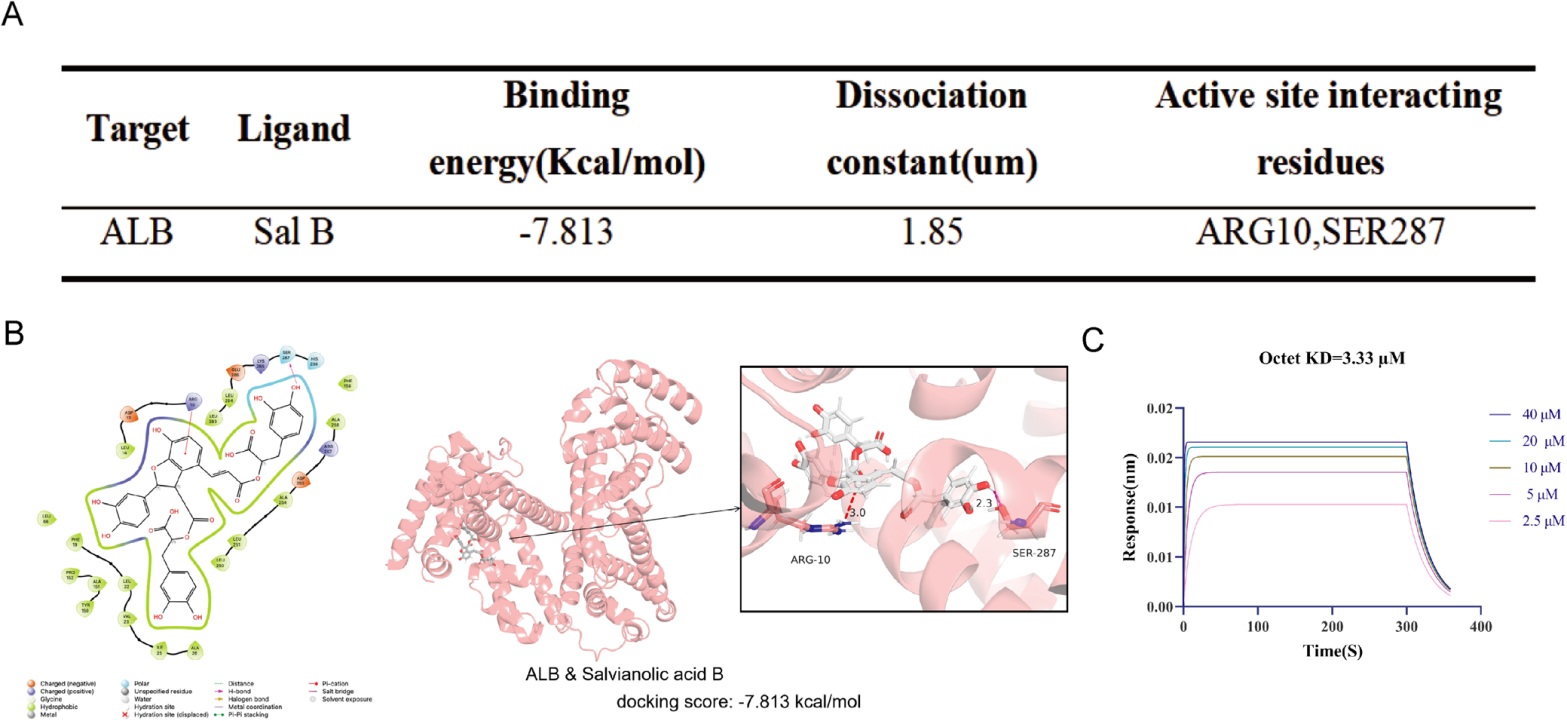
Molecular docking results of Sal B and ALB. (A) Molecular docking score table. (B) Schematic diagram of the molecular docking results of ALB and Sal B. (C) Bio‐layer interferometry (BLI)‐based measurement of the affinity between Sal B and ALB.

To explore the Sal B‐ALB interaction, their 3D structures were acquired from PubChem and the PDB, respectively. AutoDock calculated an optimal binding energy of −7.813 kcal/mol and a dissociation constant of 1.85 μM, confirming a high‐affinity interaction. The binding site analysis further identified a salt bridge (with ARG‐10) and a hydrogen bond (with SER‐287) (Figure 4A). Figure 4B (generated with PyMOL and Discovery Studio) illustrates these molecular interactions. Next, we examined the affinity of ALB proteins for Sal B by bio‐layer interferometry (BLI). Results showed that the Sal B‐ALB with a KD value of 3.33 μM (Figure 4C).

### 3.5 Molecular Dynamics Simulation Results of Sal B‐ALB Complex

**FIGURE 5:**
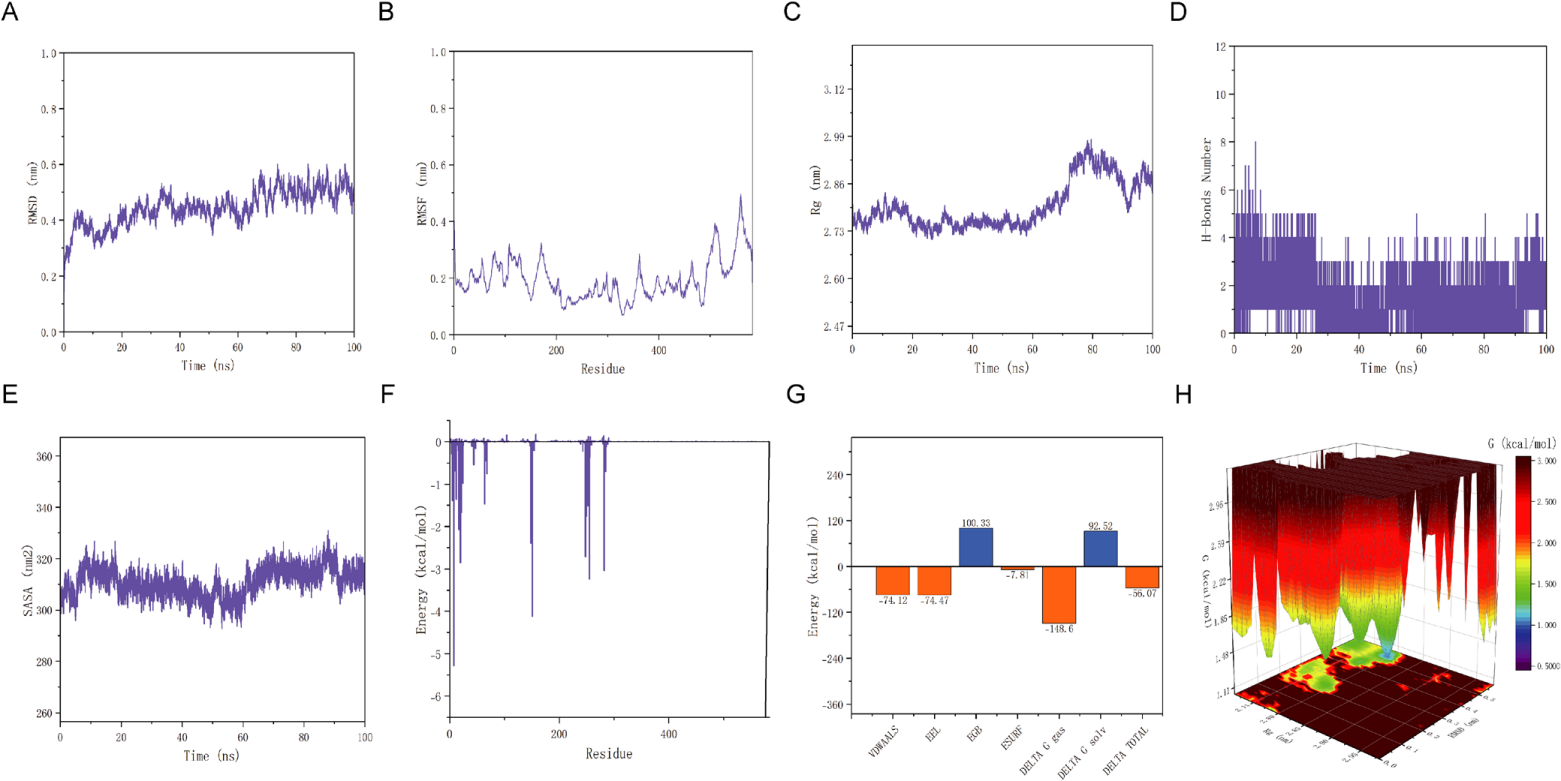
Molecular dynamics simulation of Sal B and ALB. (A) The RMSD curve. (B) The RMSF curve. (C) The Rg curve. (D) The variation of the number of hydrogen bonds during simulations. (E) The SASA curve. (F, G) The binding free energy. (H) Gibbs free energy 3D topography.

The stability of the Sal B‐ALB complex was confirmed by 100 ns of MD simulation. Post‐equilibration, the root mean square deviation (RMSD) values rapidly converged and maintained a stable plateau, reflecting high structural integrity of the complex. As depicted in Figure 5A, the RMSD values stabilized around 0.5 nm after 70–100 ns, reflecting a well‐equilibrated system. Analysis of the root mean square fluctuation (RMSF) profile revealed structural flexibility at specific residue positions, with notable peaks observed at residues 1555 and 557–564, suggesting enhanced mobility in these regions (Figure 5B). Such flexible segments may play functional roles and influence overall complex stability. The radius of gyration (Rg), an indicator of structural compactness, remained stable at approximately 2.89 nm after equilibration, indicating sustained conformational tightness (Figure 5C). Throughout the simulation, the number of hydrogen bonds between Sal B and ALB varied from 0 to 8, indicative of a stable intermolecular interaction (Figure 5D). The solvent accessible surface area (SASA) profile showed minimal variation, consistent with a stable conformational state of the complex during the simulation (Figure 5E).

The binding free energy reflects the strength of the protein‐ligand interaction over the simulation time, with lower binding free energy values indicating stronger binding affinity (Figure 5F and 5G). MM/GBSA calculations yielded a binding free energy of −56.07 kcal/mol for the Sal B‐ALB complex, indicating a highly favourable binding affinity. The free energy landscape (FEL) analysis further demonstrated that the complex predominantly adopted stable conformational states characterized by Rg values of 2.78–2.83 nm and RMSD values of 0.50–0.59 nm (Figure 5H). The stable low‐energy binding conformation of the Sal B‐ALB complex, supported by key residue interactions, suggests an agonist‐like binding mechanism for Sal B.

### 3.6 Sal B Exerts Protective Effects Against LPS‐Induced ALI

**FIGURE 6:**
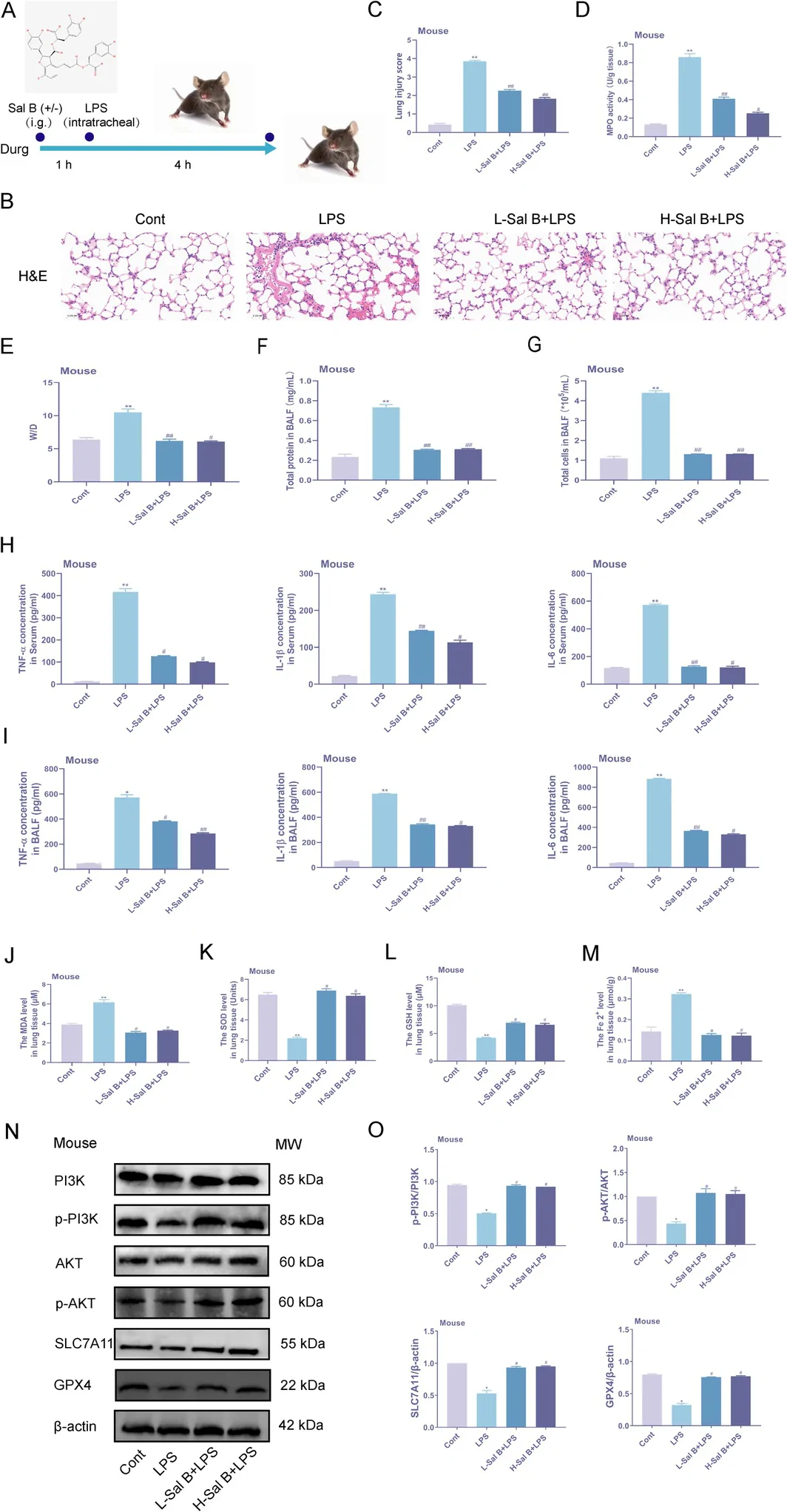
Sal B exerts protective effects on ALI mice by inhibiting the ferroptosis signalling pathway. (A) Dosing time chart of Sal B treatment in ALI mice. (B) H&E staining (×200 magnification). (C) H&E pathological score. (D) MPO activity. (E) W/D weight ratio (W/D). (F) Total protein content in BALF. (G) Total cell counts in BALF. (H) Serum concentrations of TNF‐ɑ, IL‐1β, and IL‐6. (I) Concentrations of TNF‐α, IL‐1β, and IL‐6 in BALF. (J) MDA content. (K) SOD activity. (L) GSH level. (M) Tissue Fe^2+^ level. (N) Protein expression levels of PI3K, p‐PI3K, AKT, p‐AKT, SLC7A11, and GPX4 across different groups. (O) Western blot quantification of p‐PI3K/PI3K, p‐AKT/AKT, SLC7A11, and GPX4. All data are shown as mean ± SD (*n* = 3). Compared with the control group, **p < 0.01 and *p < 0.05; compared with the model group, ^##^ *p* < 0.01 and ^#^ *p* < 0.05.

An LPS‐induced ALI model was established to evaluate Sal B (10 and 20 mg/kg) (Figure 6A). H&E results revealed that LPS caused significant lung tissue structural damage (at 4 mg/kg), including disrupted alveolar walls, alveolar haemorrhage, interstitial oedema, and inflammatory cell infiltration. Sal B treatment notably mitigated these pathological manifestations (Figure 6B). In line with these histological observations, the lung injury scores showed a marked attenuation of ALI severity in the Sal B‐treated groups relative to the LPS group (Figure 6C). MPO activity in lung homogenates was determined as an indicator of neutrophil accumulation. Sal B treatment significantly reduced MPO levels compared to the LPS group, indicating effective suppression of neutrophil recruitment into lung tissues (Figure 6D). The severity of pulmonary oedema was quantified by the lung W/D weight ratio and BALF protein concentration. Both parameters showed marked increases in the LPS‐induced ALI group compared to the control group. In contrast, Sal B treatment substantially reduced both pulmonary oedema and protein extravasation (Figure 6E,F). Consistent with these findings, Sal B pretreatment effectively inhibited the LPS‐induced elevation in total cell counts in BALF (Figure 6G). Moreover, Sal B exhibited a significant suppressive effect on the levels of key inflammatory cytokines (TNF‐α, IL‐1β, and IL‐6) in both serum and BALF of the LPS‐ALI model (Figure 6H,I), thereby protecting against sepsis‐associated lung injury.

### 3.7 Sal B Inhibits Ferroptosis in the LPS‐Induced ALI Mouse Model

Evidence highlighting the critical involvement of ferroptosis in ALI pathogenesis prompted us to evaluate the ability of Sal B to regulate this process during LPS‐induced ALI [20]. The oxidative stress status was evaluated by measuring MDA, SOD, and GSH levels in lung tissues. Compared with the control group, the LPS‐induced ALI group exhibited considerably decreased SOD and GSH activities, along with elevated MDA content. In contrast, Sal B treatment effectively reversed these alterations, leading to increased SOD and GSH levels and reduced MDA accumulation (Figure 6J–L). These results indicate that Sal B mitigates oxidative stress by suppressing lipid peroxidation and enhancing endogenous antioxidant capacity. Furthermore, intracellular Fe^2+^ levels were assessed, revealing a significant increase in the LPS‐ALI group compared to the controls. Sal B treatment profoundly attenuated this iron accumulation (Figure 6M).

To substantiate the occurrence of ferroptosis and verify the predicted role of the PI3K/AKT pathway, we performed experiments to elucidate the mechanistic link between PI3K/AKT activation and ferroptosis suppression by Sal B. The LPS group exhibited downregulated protein levels of p‐PI3K, p‐AKT, SLC7A11, and GPX4 compared to those in the controls. Conversely, Sal B administration remarkably reversed these changes (Figure 6N,O). These results collectively demonstrate that Sal B suppresses ferroptosis in LPS‐induced ALI through activation of the PI3K‐AKT pathway.

### 3.8 Sal B Suppresses Ferroptosis in BEAS‐2B Cells

**FIGURE 7:**
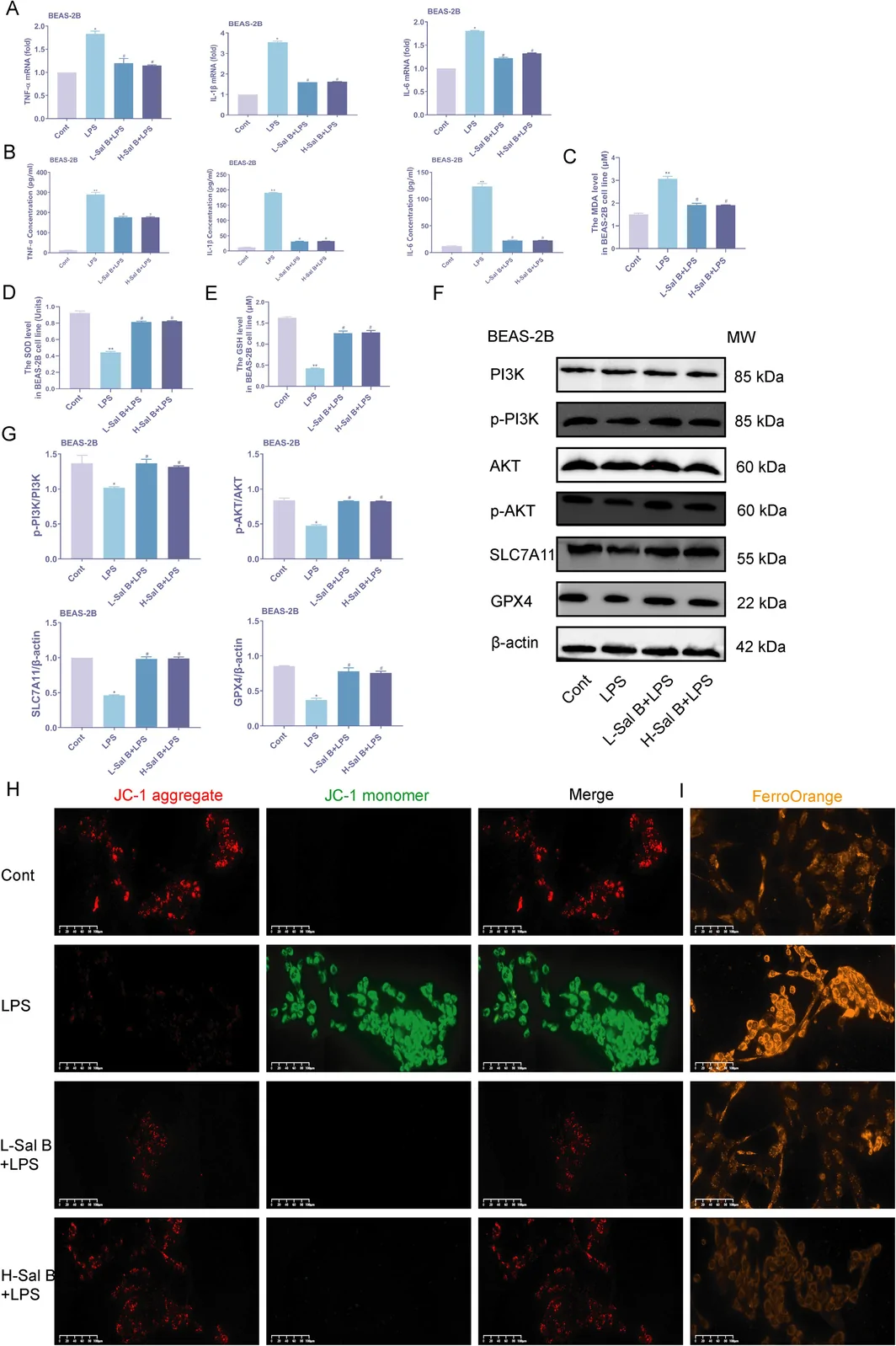
Sal B alleviates LPS‐induced BEAS‐2B cell injury. (A) Effect of Sal B on mRNA levels of inflammatory factors. (B) TNF‐α, IL‐1β, and IL‐6 concentrations measured by ELISA assay. (C) MDA content. (D) SOD activity. (E) GSH level. (F) Protein expression levels of PI3K, p‐PI3K, AKT, p‐AKT, SLC7A11, and GPX4 across different groups. (G) Western blot quantification of p‐PI3K/PI3K, p‐AKT/AKT, SLC7A11, and GPX4. (H) JC‐1 staining assay. Scale bar, 100 μm. (I) Detection of Fe^2+^ levels. Scale bar, 100 μm. All data are shown as mean ± SD (*n* = 3). Compared with the control group, **p < 0.01 and *p < 0.05; compared with the model group, ^##^ *p* < 0.01 and ^#^ *p* < 0.05.

Guided by prior evidence of Sal B's anti‐inflammatory and anti‐fibrotic effects at 20 μg/mL, we utilized BEAS‐2B cells to explore its ferroptosis‐modulating mechanisms [26]. Cells were pretreated with Sal B before LPS induction qPCR and ELISA analysis showed that Sal B significantly suppressed LPS‐induced mRNA overexpression of TNF‐α, IL‐1β, and IL‐6 (Figure 7A,B).

Assessment of oxidative stress markers revealed that LPS stimulation significantly reduced SOD and GSH levels while increasing MDA content. However, treatment with Sal B effectively attenuated oxidative stress by restoring SOD and GSH activities and suppressing MDA accumulation (Figure 7C–7E). In addition, Sal B potently counteracted the LPS‐induced downregulation of p‐PI3K, p‐AKT, SLC7A11, and GPX4 in BEAS‐2B cells (Figure 7F,G), thus demonstrating its inhibition of ferroptosis via activation of the PI3K‐AKT pathway. The dysregulation of Fe^2+^ and lipid reactive oxygen species (ROS) has been identified as a key contributor to ferroptosis initiation. Our results revealed that LPS‐induced ALI significantly enhanced intracellular Fe^2+^ accumulation, as detected by FerroOrange staining, and elevated lipid ROS levels, measured using the oxidized C11‐BODIPY probe. However, Sal B treatment robustly attenuated these pathological alterations (Figure 7H,I).

### 3.9 Sal B Mediates Protection ALI Through PI3K‐AKT Pathway Activation

**FIGURE 8:**
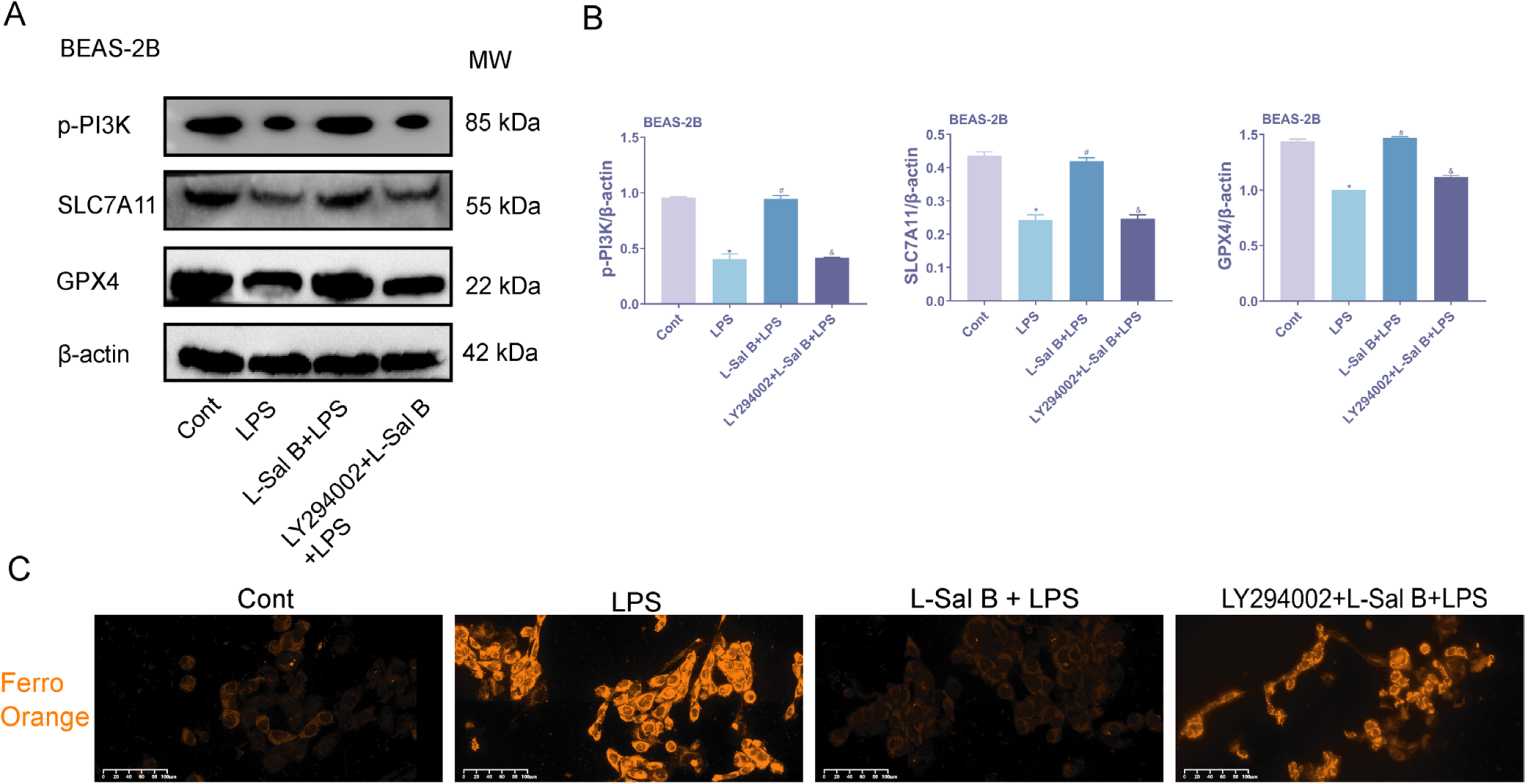
Sal B attenuates ferroptosis in LPS‐induced ALI by activating the PI3K‐AKT signalling pathway. (A) Protein expression levels of p‐PI3K, SLC7A11, and GPX4 across different groups. (B) Western blot quantification of p‐PI3K, SLC7A11, and GPX4. (C) Detection of Fe^2+^ levels across different groups. Scale bar, 100 μm. All data are shown as mean ± SD (*n* = 3). Compared with the control group, **p < 0.01 and *p < 0.05; compared with the model group, ^#^ *p <* 0.05; compared with the Sal B group, ^&^ *p <* 0.05.

As a broad‐spectrum PI3K inhibitor, LY294002 is widely utilized in mechanistic studies to interrogate the functional roles of this signalling pathway. LY294002 pretreatment sufficiently inhibited Sal B‐induced upregulation of p‐PI3K, GPX4, and SCL7A11 protein levels (Figure 8A,B). Furthermore, LY294002 significantly reversed the Sal B‐mediated downregulation of Fe^2+^ accumulation (Figure 8C). These findings comprehensively establish that Sal B‐mediated activation of the PI3K‐AKT pathway in pulmonary epithelial cells suppresses ferroptosis in LPS‐induced ALI.

### 3.10 Knockdown of ALB Attenuates LPS‐Induced Ferroptosis in BEAS‐2B

**FIGURE 9:**
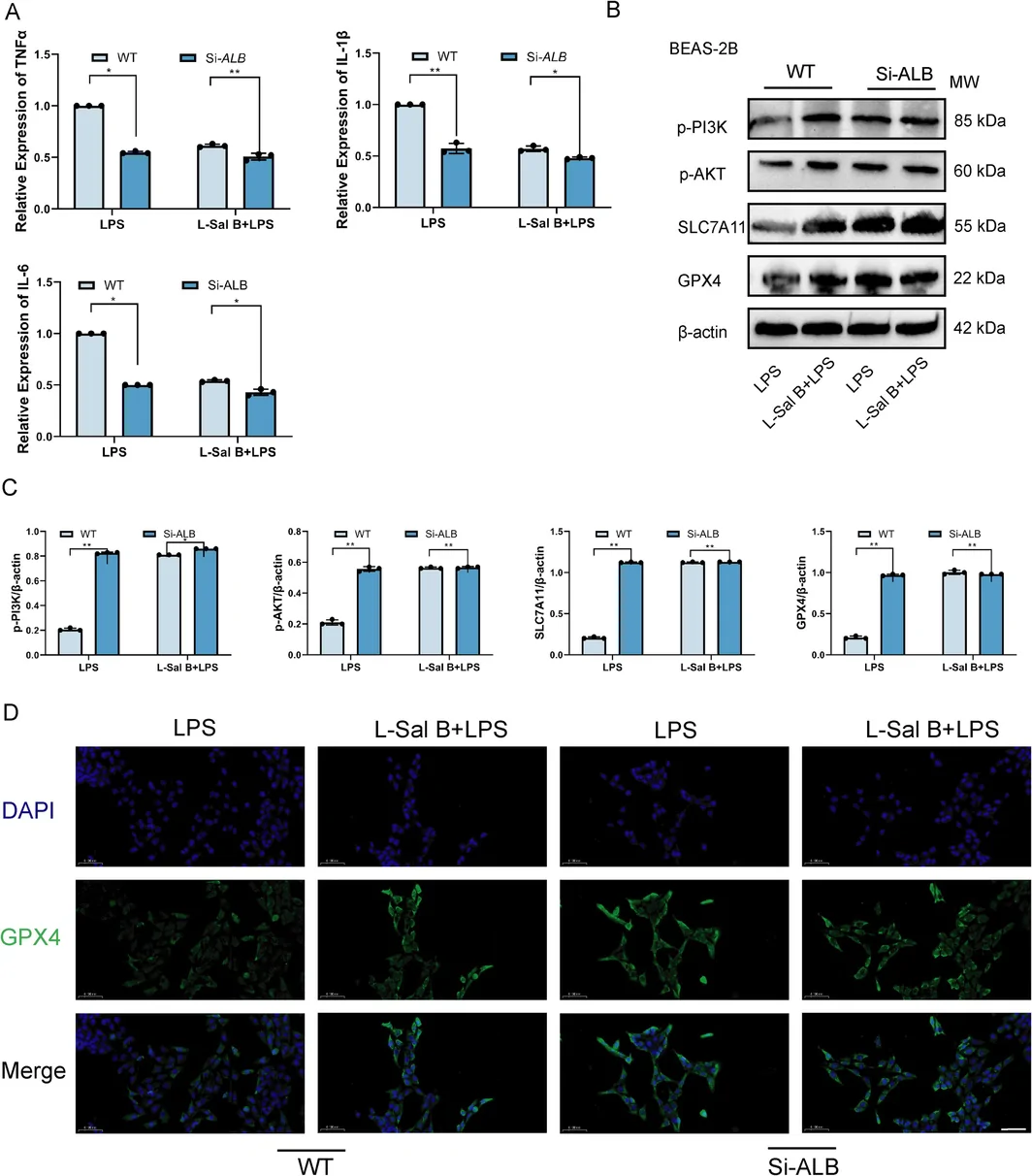
ALB silencing considerably attenuates the suppression of ferroptosis by Sal B. (A) ALB silencing obviously suppresses LPS‐induced expression of TNFα, IL‐1β, and IL‐6. (B, C) Western blot analysis and quantification of p‐PI3K, p‐AKT, SLC7A11, and GPX4 protein levels in the indicated groups. (D) Immunofluorescence staining demonstrating that ALB silencing inhibits the protective capacity of Sal B against LPS‐induced ferroptosis. Scale bar, 0.1 mm. All data are shown as mean ± SD (*n* = 3). Compared with the control group, **p < 0.01 and *p < 0.05; compared with the model group, ^##^ *p* < 0.01 and ^#^ *p* < 0.05.

We constructed a model with siRNA‐mediated ALB silencing to further test whether Sal B alleviates LPS‐induced injury through this target. Surprisingly, knockdown of ALB substantially attenuated the suppression of inflammatory factors by Sal B (Figure 9A). Si‐ALB transfection assays were carried out to characterize shifts in core ferroptosis markers based on prior findings. LPS downregulated p‐PI3K, p‐AKT, SLC7A11, and GPX4. As anticipated, Sal B rescued these aberrant phenotypes, yet this protective capacity was lost following ALB knockdown (Figure 9B,C). Immunofluorescence staining further validated that depleting ALB erased Sal B's suppression of LPS‐triggered ferroptosis (Figure 9D). Collectively, these findings demonstrate that si‐ALB substantially attenuates the Sal B‐mediated suppression of ferroptosis.

### 3.11 Transcriptome Sequencing Indicated Sal B Activated PI3K/AKT Signalling Pathway in LPS‐ALI

**FIGURE 10:**
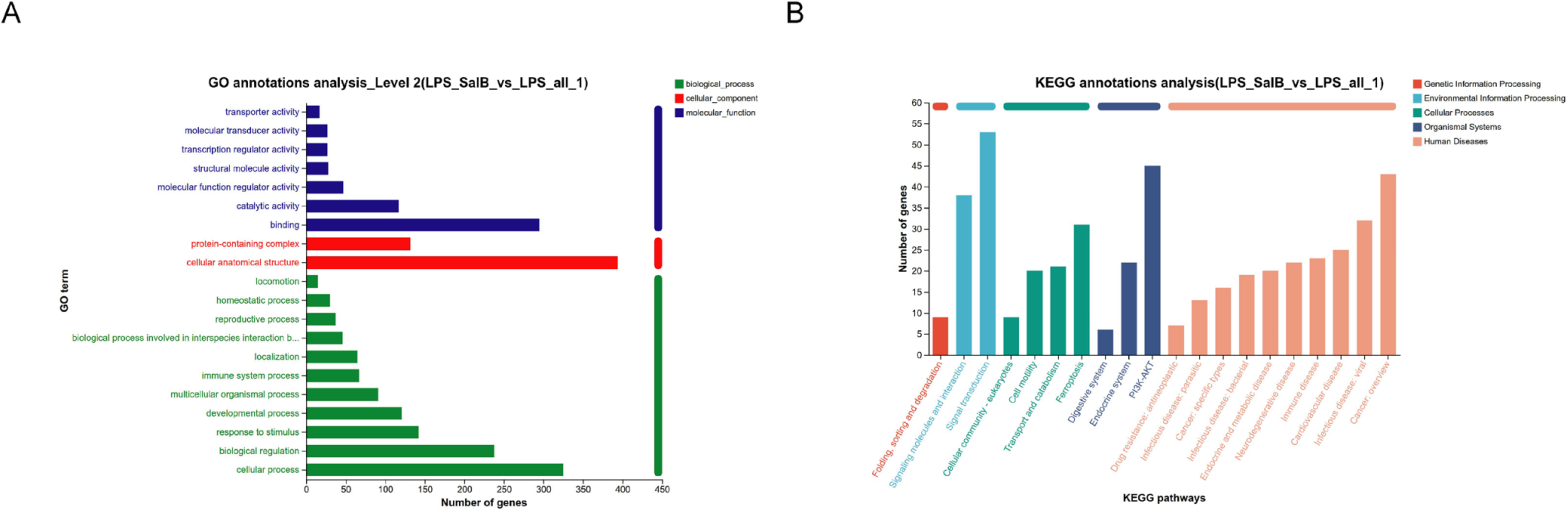
DEGs between the LPS‐Sal B and LPS groups. (A) GO enrichment. (B) KEGG enrichment.

To better validate the interaction between the Sal B and the PI3K‐AKT pathway. We conducted transcriptome sequencing of the LPS group and the Sal B‐LPS group. GO and KEGG functional enrichment analyses were performed. The three main, independent GO categories are BP, MF, and CC. BP included binding, catalytic activity, and molecular function regulator activity. CC involved protein‐containing complex and cellular anatomical structure. MF comprised cellular process, biological regulation, and response to stimulus (Figure 10A). According to the results of KEGG analysis, functional enrichment results were related to the PI3K‐AKT signalling pathway and ferroptosis (Figure 10B). Transcriptome sequencing indicated that Sal B activated the PI3K/AKT signalling pathway in LPS‐ALI.

## 4 Discussion

The pathogenesis of ALI is orchestrated by a complex cascade of inflammatory mediators and effector cells, culminating in diffuse pulmonary parenchymal damage through self‐amplifying inflammatory responses [27]. Within pulmonary tissues, intricate interactions among cells and signalling pathways form an extensive regulatory network. While traditional Chinese medical texts lack direct references to ALI or ARDS, associated symptoms such as chest tightness, dyspnoea, and cough align with traditional patterns including “chest bind”, “panting collapse”, and ‘sudden panting’ [27, 28]. In treating pulmonary disorders, TCM emphasizes heat‐clearing and detoxification, blood‐activation and stasis‐resolution, lung‐diffusing and water‐draining, and purgation techniques. Numerous active components from Chinese herbal medicine have demonstrated critical anti‐inflammatory and antioxidant activities, positioning ethnopharmacological investigation as a promising approach for novel therapeutic agents against ALI [20, 29].

Utilizing a combined computational and experimental approach, we systematically delineated the protective mechanisms of Sal B against ALI, linking its well‐defined chemical structure to its system‐level pharmacological effects. The broad pharmacological profile of Sal B is well‐established, encompassing significant antioxidant, anti‐inflammatory, and anti‐apoptotic activities [9]. In a model of myocardial ischaemia–reperfusion injury, Sal B demonstrated cardioprotective effects, mediated through activation of the PI3K/AKT signalling pathway and suppression of HMGB1 protein expression [30]. In neurological research, Sal B facilitated the neural differentiation of induced pluripotent stem cells via the PI3K/AKT/GSK3β/β‐catenin pathway, thereby conferring neuroprotective effects [31]. In an LPS‐induced sepsis rat model, Sal B alleviated lung tissue injury and reduced pro‐inflammatory cytokine levels by downregulating TRPM6 and TRPM7 expression [17]. Furthermore, studies in LPS‐induced ALI models have demonstrated that Sal B provides comprehensive protection through the concurrent suppression of apoptosis, oxidative stress, and inflammatory responses [15]. Similar to the aforementioned related research, our results clarified that Sal B significantly suppresses the expression of TNF‐α, IL‐6, and IL‐1β under LPS stimulation, thereby mediating a marked reduction in pulmonary tissue damage, inflammation, and oxidative stress injury in ALI.

Ferroptosis is a distinct, iron‐dependent form of regulated cell death characterized by the lethal accumulation of lipid peroxides [32]. Emerging evidence links ferroptosis to various forms of ALI, spanning ischaemia‐reperfusion, sepsis, radiation, drowning, and oleic acid models [33]. Triggers such as LPS, acid aspiration, or viral infection can lead to GSH depletion, inactivation or downregulation of GPX4 activity in alveolar epithelial cells, thereby disrupting redox homeostasis and causing lethal lipid peroxidation that ultimately compromises membrane integrity and pulmonary barrier function [33, 34]. The PI3K/AKT pathway functions as a central regulator of cell survival, proliferation, metabolism, and stress adaptation [18]. This pathway serves as a pivotal regulator of ferroptosis by modulating key components, including NRF2, GPX4, SLC7A11, Fe^2+^ and lipid metabolism, thereby exerting coordinated control over amino acid and lipid metabolic processes in this cell death pathway [18]. Previous research has identified coptisine as a modulator that alleviates ALI by suppressing ferroptosis in alveolar epithelial cells through the PI3K/AKT/Nrf2 signalling axis [19]. In addition, huperzine A suppressed ferroptosis through PI3K/AKT activation, upregulating the ferroptosis‐inhibitory proteins SLC7A11 and GPX4 while reducing inflammatory cytokine expression [20]. PAI‐1 deficiency has been shown to exacerbate *Pseudomonas aeruginosa* ‐induced ALI through dysregulation of the PI3K/MAPK/AKT pathway, leading to enhanced NETs formation, pyroptosis, and ferroptosis [21]. In ALI, activation of the PI3K/AKT pathway confers comprehensive protection, characterized by concerted attenuation of inflammation, suppression of apoptosis, and inhibition of ferroptosis.

Furthermore, Sal B effectively reversed ferroptosis‐related alterations, including restoration of GPX4, SLC7A11, GSH, and SOD levels, alongside reduction of Fe2+ and MDA content. Moreover, the results also demonstrated that Sal B treatment significantly enhanced p‐AKT levels, accompanied by upregulated GPX4 and SLC7A11 expression and reduced lipid ROS accumulation. This indicates that Sal B activates the PI3K/AKT pathway as a master regulatory switch, which subsequently reinforces the downstream GPX4‐mediated antioxidant defence system to fundamentally suppress ferroptosis, ultimately preserving alveolar epithelial integrity and ameliorating ALI pathology.

It is noteworthy that a wide range of natural products and synthetic compounds have been reported to exert protective effects against ALI. However, Sal B possesses several distinctive advantages over these agents.

Firstly, many previously reported ALI‐protective agents target only a single pathway, such as anti‐inflammatory or antioxidant effects alone. While the pathogenesis of ALI is exceedingly complex, involving intricate crosstalk and interactions among multiple signalling pathways and molecular networks. Thus, our study demonstrates that Sal B exerts systemic, multi‐target regulatory effects, including anti‐inflammatory, anti‐oxidative stress, anti‐apoptotic, and NLRP3 inflammasome inhibition, conferring a unique therapeutic advantage in managing the multifaceted pathophysiology of ALI.

Secondly, the mechanisms of action described for many natural products and synthetic compounds remain largely descriptive, often limited to phenotypic observations without clear molecular target identification. In contrast, the molecular targets of Sal B are progressively being elucidated. A recent study has revealed that the core mechanism lies in the potential binding of Sal B to TNFR1, which simultaneously suppresses three critical downstream pathways at their source: NF‐κB‐driven inflammatory storm, RIPK1/RIPK3/MLKL‐mediated necroptosis, and MLCK/MLC2‐triggered epithelial barrier damage [35]. This discovery advances the understanding of Sal B from broad pathway modulation to precise receptor‐targeted action, providing a solid molecular foundation for its positioning as a host‐directed therapeutic strategy.

Thirdly, many natural products with protective effects against ALI have been evaluated only at the cellular or animal level, lacking clinical safety data and thus limiting their translational potential. Sal B stands out in this regard, having completed a systematic Phase I clinical safety evaluation. A randomized, double‐blind, placebo‐controlled Phase I trial (Registration No. CTR20192236) demonstrated that Sal B injection, administered as a single dose of up to 300 mg or as 250 mg daily for five consecutive days, exhibited excellent safety and tolerability in healthy Chinese volunteers, with no serious adverse events reported [36]. This clinical safety profile establishes a robust foundation for further clinical development, which is a distinct advantage that most preclinical ALI drug candidates currently lack.

Finally, as the primary water‐soluble active constituent of *Salvia miltiorrhiza* , Sal B possesses favourable water solubility, which confers inherent advantages in formulation development. It can be formulated as an injectable preparation for rapid intervention in acute, critically ill patients, as well as an oral formulation for subsequent maintenance therapy.

However, several limitations of this study should be acknowledged. First, while our conclusions are supported by integrated bioinformatics analysis and experimental validation, more extensive cellular and animal studies are warranted to further verify these mechanisms. Additionally, while we focused primarily on GPX4 and SCL7A11, ferroptosis involves multiple systems including glutathione metabolism and iron homeostasis; thus, investigating other key regulators such as ACSL4 and FSP1 in the context of Sal B treatment represents an important future direction. Finally, given that ALI pathophysiology involves complex interactions among various immune and structural cells beyond epithelial cells, exploring whether Sal B modulates intercellular communication within the immune microenvironment to indirectly suppress ferroptosis presents a valuable avenue for further investigation.

In summary, this study establishes that Sal B protects against ALI by activating the PI3K/AKT pathway and upregulating GPX4, thereby inhibiting ferroptosis in pulmonary epithelial cells and mitigating associated oxidative stress and inflammation. These findings not only elucidate the modern molecular mechanism underlying its traditional use but also provide a rationale for developing novel anti‐ferroptosis therapies for ALI.

## 5 Conclusion

**FIGURE 11:**
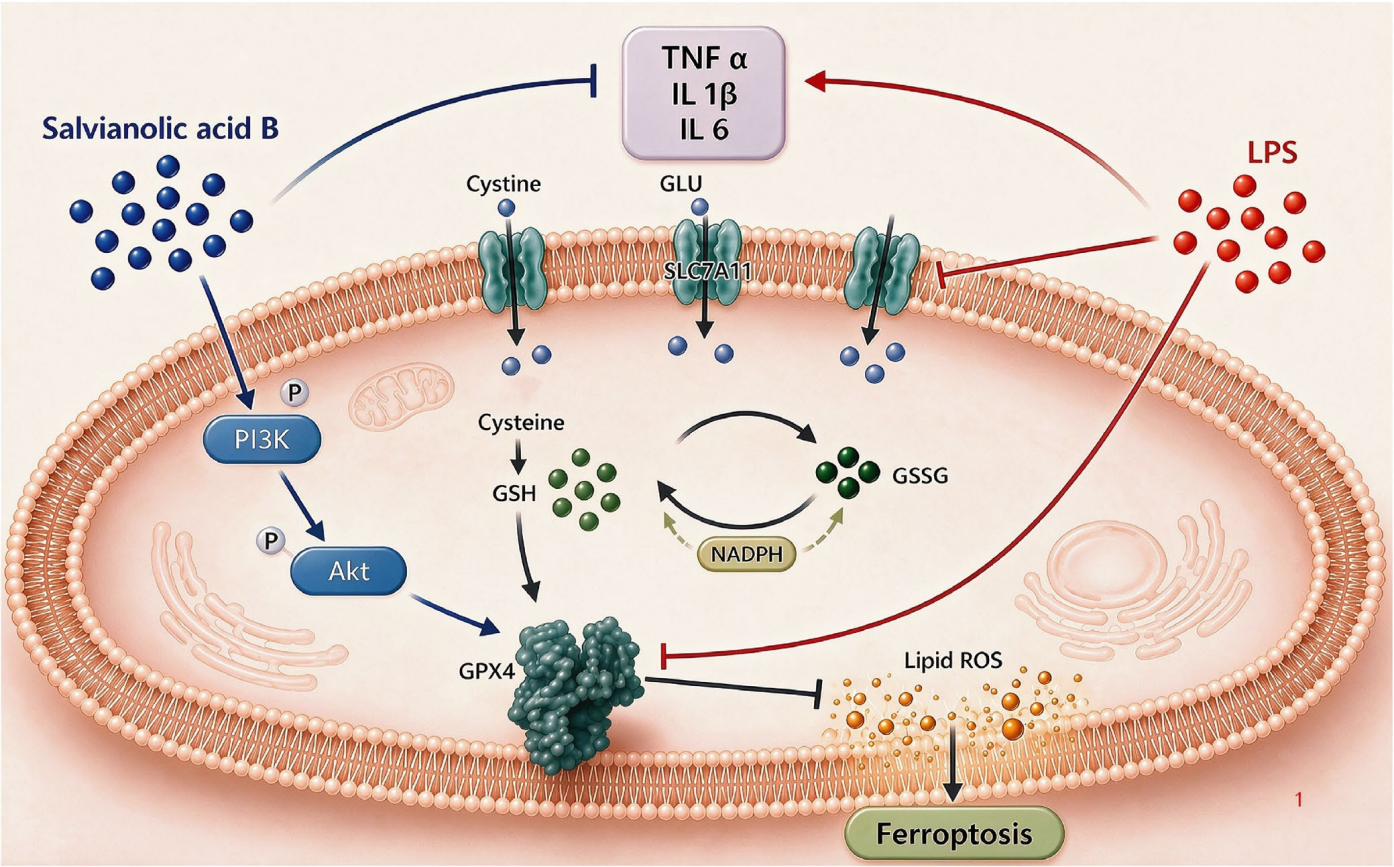
Schematic diagram of the effect of Sal B on LPS‐induced ALI.

This study demonstrates that Sal B alleviates LPS‐induced ALI by alleviating tissue injury, inflammation, oxidative stress, and ferroptosis. The protection is mediated through activation of the PI3K/AKT pathway, which elevates GPX4 and SLC7A11 expression to inhibit ferroptosis, thereby rescuing lipid ROS homeostasis (Figure 11). These findings suggest the potential of Sal B as a candidate for preventing and treating ALI/ARDS, warranting further investigation toward its clinical translation.

## Author Contributions

**Hejun Gao:** conceptualization, methodology, software, data curation, investigation, validation, formal analysis, supervision, visualization, project administration, resources, writing – original draft. **Renhui Xiong:** software, conceptualization, methodology, data curation, investigation, validation, formal analysis, supervision, visualization, writing – original draft. **Na Yue:** conceptualization, methodology, software, data curation, investigation, validation, formal analysis, supervision, visualization. **Jiaxin Wang:** conceptualization, investigation, methodology, validation, software, formal analysis, data curation, supervision, project administration, writing – review and editing, visualization, resources. **Shouyuan Tian:** conceptualization, investigation, funding acquisition, methodology, validation, visualization, writing – review and editing, software, formal analysis, project administration, data curation, supervision, resources.

## Funding

This study was supported by the Traditional Chinese Medicine (TCM) Science and Technology Innovation Project of Shanxi Province (2026kjzy006).

## Ethics Statement

All research conducted adhered to the principles of the Helsinki Declaration and the Istanbul Declaration. All protocols received approval from the Medical Ethics Committee of Shanxi Provincial People's Hospital (No. 2025–611). Institutional Animal Care and Use Committee. All experiments and animal care were performed in accordance with the guidelines outlined in the “Guide for the Care and Use of Laboratory Animals” as described by the National Institutes of Health.

## Conflicts of Interest

The authors declare no conflicts of interest.

## Supporting information


**Table S1:** Primers used in qPCR.

**Table S2:** Topological Parameters of the Top 30 Genes in the PPI network.

## Data Availability

The data that supports the findings of this study are available in the supporting information of this article.
