# Supplementary material for: Salvianolic Acid B Inhibits Ferroptosis in Acute Lung Injury: Network Analysis and Experimental Validation
Source: J Cell Mol Med. 2026 Sep 3;30(17):e71344. doi: 10.1111/jcmm.71344 (PMC13542564; doi:10.1111/jcmm.71344)
Supplement: Supplementary file 1 [file JCMM-30-e71344-s001.docx]

**Supplementary Table 1 Primers used in qPCR**

| Gene name | Forward primer sequence (5′-3′) | Reverse primer sequence (5′-3′) |
| --- | --- | --- |
| GAPDH | GCCTCGTCTCATAGACAAGATG | CAGTAGACTCCACGACATAC |
| TNF-α | CGTCAGCCGATTTGCTATCT | CGGACTCCGCAAAGTCTAAG |
| IL-1β | CTATGTCTTGCCCGTGGAG | CATCATCCCACGAGTCACA |
| IL-6 | AGTTGCCTTCTTGGGACTGA | TCCACGATTTCCCAGAGAAC |
