# Supplementary material for: Salvianolic Acid B Inhibits Ferroptosis in Acute Lung Injury: Network Analysis and Experimental Validation
Source: J Cell Mol Med. 2026 Sep 3;30(17):e71344. doi: 10.1111/jcmm.71344 (PMC13542564; doi:10.1111/jcmm.71344)
Supplement: Supplementary file 2 [file JCMM-30-e71344-s002.docx]

**Supplementary Table 2 Topological Parameters of the Top 30 Genes in the PPI network**

| name | Betweenness |
| --- | --- |
| ALB | 4393.078372 |
| TNF | 3607.611482 |
| CTNNB1 | 2240.040934 |
| CASP3 | 1562.556551 |
| CTSD | 1163.790210 |
| HRAS | 1049.670623 |
| ITGB1 | 933.5976362 |
| APP | 930.6187508 |
| TYMS | 798.8987748 |
| IL10 | 746.8569911 |
| HSP90AB1 | 586.9645835 |
| NPC2 | 578.0000000 |
| GART | 514.8329866 |
| IL2 | 511.2013156 |
| ACE | 477.6446237 |
| GRB2 | 476.1808832 |
| AKR1B1 | 460.0193938 |
| DHFR | 396.0537734 |
| MMP9 | 384.5061110 |
| MTOR | 357.0570985 |
| CHAT | 345.5067529 |
| CANX | 329.4103527 |
| CCND1 | 328.2026926 |
| JUN | 305.6927835 |
| RUVBL1 | 300.9798091 |
| ABCB1 | 296.7058223 |
| PPP1CA | 296.2656106 |
| GALK1 | 291.6833333 |
| ACADS | 291.0000000 |
| HSD11B1 | 290.0000000 |
